# Analysis of Human Degraded DNA in Forensic Genetics

**DOI:** 10.3390/genes16111375

**Published:** 2025-11-11

**Authors:** Irena Zupanič Pajnič

**Affiliations:** Institute of Forensic Medicine, Faculty of Medicine, University of Ljubljana, Korytkova 2, 1000 Ljubljana, Slovenia; irena.zupanic@mf.uni-lj.si; Tel.: +386-1-543-72-15

**Keywords:** degraded DNA, DNA fragmentation, DNA preservation, human identification, identity-informative SNP

## Abstract

Upon an organism’s death, enzymatic DNA repair ceases, exposing the genome to destructive factors such as free cellular nucleases and proliferating microorganisms, which can cause DNA loss. DNA preservation is highly dependent on environmental conditions, and less favorable environments accelerate degradation. Despite this, advanced extraction and analytical methods now enable the study of poorly preserved and degraded DNA. DNA typing is a foundation of forensic genomics, enabling the identification of individuals and the individualization of biological evidence through the generation of unique genetic profiles. Although DNA is relatively stable, environmental exposure initiates its degradation into progressively shorter fragments, complicating analysis. The extent of DNA preservation in biological evidence depends on numerous factors, and this review focuses on the environmental factors—including temperature, humidity, ultraviolet radiation, pH, chemical agents, and microbial activity—as the most influential variables. In samples with degraded DNA, the maximum amplicon length achievable through polymerase chain reaction (PCR) is inherently limited. This review discusses genetic markers and analytical strategies improvements that enable the examination of highly degraded samples, particularly when conventional short tandem repeat (STR) typing fails. In these situations, successful identification requires targeting short DNA fragments, which are more likely to persist. Single-nucleotide polymorphisms (SNPs) are a valuable alternative, as their high allelic variability and short amplicon requirements make them more amenable to amplification from fragmented templates than STRs. Advances in next-generation sequencing (NGS) technologies have further enhanced this capacity by enabling high-resolution SNP profiling, thereby improving outcomes in challenging forensic cases.

## 1. Introduction

Molecular genetic DNA analysis has revolutionized forensic science, becoming an indispensable tool for the accurate identification of unidentified human remains and the individualization of biological evidence from crime scenes [1]. The integration of DNA technology into forensic practice has fundamentally transformed investigative capabilities, enabling precise human identification even when traditional methods fail. These techniques are universally regarded as the gold standard in forensic casework due to their high discriminatory power and statistical robustness [2].

The foundation of modern forensic DNA analysis lies in nuclear autosomal DNA, which serves as the primary marker for identification purposes due to its biparental inheritance and high mutation rate. This preference is attributed to the exceptional polymorphism found in autosomes, resulting from meiotic recombination events and codominant inheritance patterns that provide extraordinary individual discrimination [3]. The human genome contains numerous polymorphic loci that have been systematically characterized for forensic application, with international standardization ensuring reproducibility across laboratories [4].

Forensic applications predominantly focus on analyzing length polymorphisms in short tandem repeat (STR) regions, which are distributed throughout the nuclear genome [5]. The effectiveness of STR profiling, however, depends on how well the DNA in the biological sample has been preserved. Under optimal conditions, STR analysis can successfully generate complete genetic profiles for human identification through multiplex polymerase chain reaction (PCR) amplification of multiple loci simultaneously [4]. Nevertheless, forensic scientists frequently deal with challenging samples where DNA integrity is compromised through various degradation processes that affect amplification efficiency [6].

Skeletal remains represent particularly difficult specimens due to the complex nature of DNA extraction from the bone matrix and the frequent presence of PCR inhibitors that copurify with nucleic acids [7]. The mineralized composition of bone and teeth provides some protection against environmental degradation but also presents substantial challenges for DNA recovery [8]. Similarly, archived tissues, especially those preserved in formalin, present substantial obstacles for genetic analysis due to protein-DNA crosslinking and nucleic acid fragmentation. Formalin fixation induces DNA degradation through methylene bridge formation, and prolonged exposure significantly intensifies fragmentation patterns [9].

Biological evidence exposed to unfavorable environmental conditions often suffers extensive DNA damage through hydrolytic, oxidative, and enzymatic processes [10]. Such damage results in low quantities of heavily degraded DNA that prevent complete STR profile generation due to the preferential amplification of shorter fragments in compromised samples [11]. Environmental factors, including temperature fluctuations, humidity, ultraviolet (UV) radiation, and microbial activity, collectively determine DNA preservation quality, creating complex degradation patterns that challenge conventional analysis [12].

To address these limitations, mitochondrial DNA (mtDNA) analysis has historically been employed for challenging forensic samples [13,14]. The high copy number per cell (ranging from hundreds to thousands of copies) and circular structure of mtDNA enhance its persistence compared to nuclear DNA [15,16]. However, maternal inheritance patterns and the consequent lack of individual discrimination limit its forensic applicability to exclusion purposes rather than identification [14]. The absence of recombinant events in mtDNA genome evolution means that mtDNA represents a single linked locus, reducing its discriminatory power compared to multiple independent autosomal markers [17].

Recent advances in forensic genetics have introduced sophisticated solutions for analyzing degraded DNA through next-generation sequencing (NGS) technologies, also referred to as massive parallel sequencing (MPS) [18,19]. Single-nucleotide polymorphisms (SNPs) with identity-informative SNPs (iiSNPs) have emerged as powerful markers, either supplementing or replacing traditional STR analysis in compromised samples [20]. Characterized by short amplicons typically under 150 base pairs, SNPs can be efficiently analyzed using NGS platforms that simultaneously detect numerous markers [21]. Their biallelic nature and location throughout the nuclear genome provide complementary discriminatory power to STRs, while their minimal length requirements make them particularly suitable for highly degraded samples where conventional STR typing fails [22]. In Table 1, forensic genetic markers (STRs, iiSNPs, and mtDNA) are compared.

The development of targeted enrichment methods and optimized library preparation protocols has further enhanced recovery of genetic information from compromised samples [29,30,31]. These technological advancements, combined with improved DNA extraction techniques specifically designed for degraded samples, have progressively expanded the limits of forensic DNA analysis [32].

This review examines the complex interplay between environmental factors, DNA degradation mechanisms, and the evolving analytical strategies employed in forensic genetic analysis of compromised samples. We discuss the theoretical foundations of DNA degradation processes, practical considerations for sample handling, and the implementation of novel genetic markers and sequencing technologies that continue to expand the boundaries of forensic identification capabilities in modern forensic practice.

## 2. Environmental Factors Affecting DNA Preservation

The preservation of DNA postmortem is a fundamental concern across numerous scientific disciplines, from forensic investigation to paleogenomics. Although DNA is a remarkably stable molecule within the protected environment of a living cell, its structural integrity is inevitably compromised following death, initiating a complex and continuous process of decay [10]. DNA degradation is affected by a dynamic interplay of intrinsic biochemical reactions and extrinsic environmental conditions [33]. A comprehensive understanding of these variables is therefore critical for developing effective sample recovery protocols, optimizing laboratory analytical techniques, and ensuring the accurate interpretation of genetic results derived from degraded biological samples [34,35].

The key mechanisms for initial postmortem DNA damage are enzymatic. In life, a sophisticated array of cellular repair mechanisms involved in DNA replication continuously identifies and corrects lesions in the DNA molecule [36]. Upon death, these systems fail, leaving the DNA unprotected. In addition, metabolic activity generates reactive oxygen species (ROS), which oxidize bases and sugars, leading to miscoding lesions and strand breaks that also require repair [37]. After death, not only replication but also metabolic ROS production ceases, meaning that these sources of endogenous DNA damage are no longer active. At the same time, other post-mortem chemical, microbial, and environmental factors become the dominant contributors to DNA degradation [38]. The first wave of destruction often comes from endogenous nucleases—enzymes present within the cell itself—that become activated and begin to cleave the DNA backbone. This is followed by exogenous enzymatic attack from microbes that colonize the decomposing remains and crime case biological traces, releasing their own nucleases that further fragment the genetic material [33]. The most visible outcome of this process is fragmentation, where the long, continuous strands of DNA are cleaved into much shorter pieces, typically ranging from 100 or less to 500 base pairs, due to the breakdown of both covalent bonds in the sugar-phosphate backbone and the hydrogen bonds holding the double helix together [39].

Alongside this enzymatic degradation, slower but persistent chemical processes continue the damage. Hydrolytic reactions, driven by water molecules, attack the DNA, leading to the loss of nitrogenous bases (a process known as depurination) and the deamination of cytosine to uracil. Oxidative damage, inflicted by free radicals and reactive oxygen species, causes base modifications, sugar alterations, and additional strand breaks. Furthermore, exposure to UV radiation can induce the formation of cyclobutane pyrimidine dimers, which distort the DNA helix and block polymerase activity during subsequent analysis [10,12]. It is only under exceptional conditions that these processes are markedly slowed. Rapid dehydration, as occurs in arid environments, or instant freezing, as in permafrost or glaciers, can inhibit microbial activity and dramatically reduce the rate of hydrolytic and oxidative reactions, thereby preserving DNA for centuries or even millennia.

The specific environmental conditions in which remains are deposited ultimately determine DNA survival. In addition, the duration of exposure is a critical variable, with prolonged intervals generally leading to accelerated and more comprehensive degradation [40,41,42]. Post-recovery handling is also a significant factor; improper collection or storage of biological evidence or human remains can introduce modern contaminants, cross-contamination between samples, or with the collector’s DNA, or cause further damage, severely impacting the quantity and quality of recoverable DNA [43].

Among environmental factors, temperature is considered the most influential [44,45,46]. It acts as a primary regulator, controlling the kinetic energy of all atoms and molecules and thus dictating the rate of every chemical reaction, including hydrolysis and oxidation. It also directly governs the metabolic rate and replication speed of decomposer microorganisms. Even a modest increase of 2 °C in ambient temperature has been shown to measurably impact the stability of nuclear DNA, and a 10 °C rise can double or triple the rate of destructive chemical processes [47,48]. This explains the stark contrast between the exceptional preservation seen in remains recovered from constant cold environments, such as permafrost, and the near-total degradation observed in contexts characterized by high heat and humidity [49,50].

Moisture is another primary agent of decay, acting as both a necessary reactant in hydrolysis and a prerequisite for microbial life. Consequently, environments with high water activity are extremely detrimental to DNA preservation. The pH of the depositional environment, often a consequence of moisture and microbial metabolism, is equally critical. Highly acidic or alkaline conditions catalyze chemical degradation, while a neutral to slightly alkaline pH is generally most favorable for preservation, as it can suppress the activity of many degradative nucleases [8,51]. The impact of soil pH is profound; research has demonstrated that the decomposition of soft tissue can proceed up to three times faster in acidic soils compared to their alkaline counterparts [52].

For buried remains, the geochemical composition of the soil or sediment is a major determinant of preservation. DNA molecules can adsorb onto certain mineral surfaces, such as clays and hydroxyapatite, which can shield them from enzymatic attacks and slow hydrolytic decay [53]. Conversely, soils rich in organic matter typically support a much greater abundance and diversity of decomposer microbes. Furthermore, organic matter itself can release humic and fulvic acids, which are potent inhibitors of the polymerases used in PCR, complicating downstream genetic analysis even if some DNA persists [54,55]. Microbial activity is a dual threat; it directly degrades DNA through enzymatic cleavage and indirectly damages it by altering the burial microenvironment. The metabolic byproducts of bacteria and fungi, including various organic acids, can significantly acidify the local environment, thereby accelerating chemical hydrolysis [56].

Other environmental forces also contribute to cumulative damage. The presence of oxygen facilitates oxidative damage through the formation of free radicals. Physical forces, such as repeated freeze–thaw cycles that cause ice crystal formation, mechanical pressure from overlying sediment, or the pervasive growth of plant roots through bone, can all cause physical shearing of DNA strands into ever-shorter fragments [57,58]. Deeper burials often provide a more stable environment, buffering against temperature fluctuations, reducing oxygen exposure, and eliminating the damaging effects of UV radiation [8,59]. In Table 2, the main degradation factors influencing DNA preservation are summarized.

Despite the formidable array of challenges posed by the environment, technological innovations in molecular biology have dramatically improved the prospects for recovering genetic information from highly degraded sources. A pivotal advancement was the development of silica-based extraction methods, which efficiently bind DNA fragments while removing a wide range of potent environmental inhibitors, such as humic acids, that would otherwise paralyze enzymatic reactions in the laboratory [60,61]. Perhaps an even more significant shift has been the change in analytical strategy. Instead of relying on long, intact DNA sequences, scientists now focus on shorter, more resilient genetic markers, such as SNPs. The advent of NGS technologies allows for the simultaneous recovery and analysis of thousands of SNPs, enabling the reconstruction of reliable genetic profiles from even the most minimal, damaged, and contaminated sources [62].

## 3. Strategies for Successful DNA Acquisition from Compromised Forensic Samples

The acquisition of sufficient DNA from challenging biological evidence represents a foundational step in the forensic genetic analysis pipeline, determining the ultimate success or failure of obtaining a reliable genetic profile. Samples exposed to environmental insults, such as skeletal remains, decomposed tissue, or touch evidence, often contain minimal quantities of DNA that are highly degraded and contaminated with PCR inhibitors. Because of PCR inhibitors, the selection and application of optimized extraction and purification protocols are not merely beneficial but essential for downstream analytical success [32,63,64]. PCR inhibitors interfere with amplification through several mechanistic routes. Some inhibitors, such as melanin and hematin, interact directly with DNA polymerase and reduce its catalytic activity, leading to decreased amplification efficiency and allele dropout [65,66]. Other compounds, including humic and fulvic acids or tannins, non-specifically bind nucleic acids or primers, sterically blocking primer annealing or extension [65,67]. Certain inhibitors can chelate essential cofactors, notably Mg^2+^, thereby disrupting enzyme–substrate interactions [66]. In addition, some substances quench fluorescent dyes or probes, producing apparent signal loss even when amplicons are generated [65,67].

The choice of extraction methodology is arguably the most critical factor influencing the quality of the recovered genetic material. Extensive research has demonstrated that silica-based methods, particularly those utilizing paramagnetic bead technology, consistently outperform traditional organic extraction for compromised samples. These advanced techniques function by binding DNA to silica surfaces in the presence of high concentrations of chaotropic salts or alcohol, which disrupt cellular structures and denature proteins. This process allows for efficient washing steps that effectively co-purify DNA while removing a vast array of potent PCR inhibitors, including humic acids, melanin, tannins, and hematin, which are prevalent in forensic samples [11,60,68]. Specialized inhibitor removal strategies incorporated in some commercial kits—such as polymeric resins, proprietary buffers, or adsorption columns—can selectively eliminate humic acids, pigments, heme derivatives, and other compounds that interfere with polymerase activity, primer annealing, or fluorescent detection. By combining DNA binding, stringent washing, and targeted inhibitor removal, these approaches increase the likelihood of successful PCR amplification even from highly degraded or inhibitor-rich forensic or ancient DNA samples [65,66,67]. The high efficiency of magnetic bead-based purification systems for inhibitor removal and DNA recovery from a wide range of substrates—including bone, teeth, and formalin-fixed tissues—has been robustly confirmed through numerous forensic DNA studies [32,69,70,71,72].

Given the typically low copy number of endogenous DNA in such samples, the constant risk of contamination with exogenous modern DNA is a paramount concern. The field has therefore adopted rigorous anti-contamination protocols, many of which were pioneered in ancient DNA research. These measures are designed to prevent the introduction of contaminant DNA at every stage of analysis. Essential practices include the physical separation of pre- and post-PCR laboratory areas, the implementation of a strict unidirectional workflow (moving from clean pre-PCR spaces to post-PCR areas), and the rigorous decontamination of workspaces and equipment using sodium hypochlorite (bleach) and UV irradiation [39,73,74]. The processing of extracts and the setup of PCRs should be conducted in dedicated, positive-pressure laboratories equipped with HEPA-filtered hoods, and personnel must wear full protective clothing, including masks, face shields, and gloves [75,76,77].

To monitor any contamination introduced during the laboratory process, the inclusion of multiple negative controls is mandatory. Both negative extraction controls (blank samples processed alongside the evidence) and negative PCR controls (reaction mixtures without template DNA) must be processed in parallel with the casework samples. The implementation of this comprehensive control system allows for the detection of contamination at its source, whether from reagents, surfaces, or personnel [78,79]. Furthermore, a highly recommended practice is to genotype all laboratory personnel who have handled the samples, creating a reference database to identify potential sources of contamination. For the highest level of confidence, replication is key: extracting DNA from the same sample at least twice, obtaining concordant genetic profiles from both extracts, provides strong authentication of the results. If contamination is detected in negative controls, the corresponding results must be considered invalid, and the analyses repeated from a new extraction. In addition, the use of a multitube approach, where multiple independent amplifications are performed, further strengthens the reliability of the obtained genetic profiles [39,79].

Following extraction, the accurate quantification of DNA is crucial for successful amplification. Modern multiplex quantitative PCR (qPCR) kits provide more information than mere concentration. They simultaneously assess the total human DNA quantity, provide a degradation index (e.g., the ratio of a large target amplicon to a small one), and measure the presence and relative amount of PCR inhibitors in the sample. This tripartite data is indispensable for making informed decisions on how to proceed with the analysis [80,81].

The information obtained from quantification directly guides the selection of the most appropriate genotyping strategy. For samples exhibiting a high degradation index, conventional STR amplification may fail due to the inability to amplify longer fragments. In such cases, alternative marker systems are required. SNP panels, which utilize very short amplicons (<100–150 bp), are often far more successful for genotyping highly degraded DNA. The integration of DNA quantification and degradation assessment data thus allows forensic geneticists to strategically choose between standard STR kits or SNP panels, and to optimize PCR cycling conditions and input DNA volume to maximize the chance of generating a complete and interpretable genetic profile [81,82]. This data-driven, tailored approach is essential for pushing the boundaries of forensic genetics and recovering genetic information from the most challenging and degraded evidentiary materials.

## 4. Standard Human Identification Analysis

In forensic investigations, the established standard for personal identification is the genetic profiling of autosomal STR markers [1]. They exhibit length-based polymorphisms, which allow forensic experts to distinguish and identify individuals. The variability of STR markers stems from differences in the number of repeated motifs, known as length polymorphisms, which grant them significant discriminative power and are crucial for their reliability in forensic applications. A high degree of polymorphism is a result of a mutation rate that is higher than that of other genomic regions, primarily due to replication slippage during DNA synthesis, making STR loci particularly informative for distinguishing between individuals [83]. This is crucial for tasks such as verifying kinship, identifying victims of mass disasters, identifying missing persons, determining the identity of war victims, and the individualization of biological traces in crime cases [84,85]. The recovery of biological trace evidence begins at the crime scene, where the proper collection, preservation, and avoidance of contamination are paramount, as improper handling can render a sample useless for subsequent DNA analysis [86].

STR markers are highly polymorphic, making them some of the most uniquely informative regions of the human genome. The power of discrimination is so high that the probability of two unrelated individuals sharing a full DNA profile across a standard set of markers can be less than one in a billion, effectively guaranteeing uniqueness [4]. To quantify the discriminating power of a given STR marker set, it is possible to compute the Probability of Identity (PID), which estimates the chance that two unrelated individuals will, by coincidence, share an identical multilocus genotype. A more conservative measure, PIDsib, considers the elevated probability that two full siblings may share identical genotypes and thus provides a stricter upper bound when relatedness cannot be excluded [87,88]. In practical terms, when 15–24 highly polymorphic STR loci are analyzed (as in commonly used forensic amplification kits), PID values typically fall below 10^−15^, while PIDsib values are usually around 10^−4^ to 10^−6^, still providing very strong discrimination even among close relatives [88]. To quantify the strength of DNA evidence, the Likelihood Ratio (LR) is used, and it compares the probability that a DNA sample originates from the individual of interest with the probability that it comes from an unrelated random individual from the relevant population. An LR greater than 1 supports the hypothesis that the sample belongs to the target individual, while an LR less than 1 supports the alternative. The combined power of 16 to 24 STR loci allows LR values for unrelated individuals to reach extremely high orders of magnitude, effectively confirming identity. For close relatives such as siblings or parent–child pairs, the LR is lower due to the sharing of alleles, but still provides valuable statistical evidence. Accurate LR calculation requires population-specific allele frequency databases to avoid bias, and probabilistic genotyping software is often employed for complex or degraded samples, particularly in the presence of DNA mixtures. The LR complements PID and PIDsib by providing a likelihood-based measure of how strongly DNA evidence supports one hypothesis over another [89]. In Table 3, calculation methods for STR markers are summarized, and in Table 4, approximate LR values for 16–24 STR loci are shown.

STR markers consist of short, tandemly repeated sequences, typically four nucleotides long, which are referred to as tetranucleotide repeats. The choice of tetranucleotide repeats, as opposed to di- or tri-nucleotide repeats, is intentional; they exhibit lower rates of stutter artifacts during PCR amplification, which simplifies data interpretation and improves the accuracy of genotyping [1]. At each STR locus, an individual has two alleles: one inherited from the mother and one from the father. The number of these repeats varies among individuals, providing the basis for differentiation. Numerical values corresponding to the number of repeat units are assigned to these alleles.

Using the PCR, DNA is amplified; this typically requires 0.5–1 ng of DNA for optimal results, as amplification below 0.1 ng may produce stochastic effects like allele dropout, which occurs when an allele fails to amplify [90]. Furthermore, the analysis of low-template or degraded DNA presents significant challenges, such as increased stochastic effects and peak height imbalance, requiring specialized interpretation protocols [69]. By analyzing a larger panel of STR markers—commonly through multiplex kits that simultaneously amplify 16 to 24 loci (or even more), each ranging from 100 to 450 base pairs in length—it becomes possible to distinguish between all individuals, except monozygotic twins who share identical genetic information [91]. Following PCR amplification, capillary electrophoresis (CE) is used to separate the STR fragments based on size [92]. This process yields an individual-specific allelic pattern, or DNA profile, which can then be used to compare samples and assess common origin or familial relationships [93,94,95].

In criminal investigations, DNA profiles from biological traces are systematically compared to those of suspects, victims, or entries in databases. The interpretation of mixed DNA profiles, which originate from two or more individuals, remains one of the most complex tasks in forensic genetics, requiring sophisticated probabilistic genotyping software to deconvolute the contributors and calculate valid likelihood ratios [96]. The development of comprehensive STR databases across countries has significantly improved the ability to identify perpetrators over time [5]. Both forensic and missing-person DNA databases are critical for recognizing suspects, linking evidence from separate cases, and guiding investigations toward resolving crimes or locating missing individuals [4]. The efficacy of these databases relies on international cooperation and standardization of typing kits to ensure profiles are compatible across borders, an effort led by organizations like the European Network of Forensic Science Institutes (ENFSI) and the Scientific Working Group on DNA Analysis Methods (SWGDAM) [97].

When identifying an unknown body, a comparison of biological material can be made either with the deceased’s personal items (a toothbrush, razor, etc.) or with DNA from relatives. The common origin can be confirmed if the profiles from personal items match the biological sample of the unknown deceased, and the strength of this evidence is quantified using a LR [90]. The statistical framework for interpreting DNA evidence is based on population genetics, and it requires using appropriate allele frequency databases that are representative of the relevant population to avoid biased calculations [98]. The presence of shared alleles at each STR locus allows for the statistical determination of biological relatedness when a close relative, such as a child or parent, is available for comparison. For more distant relatives, the likelihood of relatedness decreases due to recombination events. In such scenarios, Y-chromosome STR markers can be analyzed if paternal-line relatives are available, since the Y chromosome is transmitted unchanged from father to son, making it identical among male descendants of a common paternal ancestor [93]. Similarly, mtDNA analysis can enhance the probability of establishing relatedness for maternal-line relatives [94].

## 5. Analysis of Degraded DNA

The analysis of highly fragmented DNA presents one of the most significant challenges in forensic genetics, whether for crime scene evidence or the identification of unrecognizable human remains. These challenges are compounded not only by the difficulty of obtaining complete genetic profiles but also by an increased susceptibility to contamination from microbial DNA or exogenous human DNA introduced during handling [99]. Microbial contamination can cause the analysis to fail, while contamination with foreign human DNA may lead to erroneous conclusions. Furthermore, severely degraded DNA is highly susceptible to secondary transfer, where DNA is moved from one surface to another, creating a high potential for misleading results that can misdirect an investigation [100]. In kinship analyses, DNA fragmentation drastically increases error rates—primarily through allele dropouts—which in turn severely reduces the reliability of statistical relatedness estimates [101,102,103]. A human cell contains roughly 6.6 pg of genomic DNA, and when working with samples containing less than 100 pg (approximately 15–17 cells), analysts frequently encounter allele dropout and incomplete or partial STR profiles; in the worst cases, STR typing fails, preventing the generation of any usable profile [90].

For the most challenging forensic samples—those with minimal DNA quantity, high degradation, and the presence of PCR inhibitors—STR typing is often simply not possible. MtDNA analysis has historically been employed for such highly degraded samples [13,14]. The utility of mtDNA stems from its high copy number per cell and circular structure, which often allows it to persist longer than nuclear DNA in deteriorating conditions, making it a valuable target when nuclear analysis fails [80]. However, it is crucial to note that mtDNA is maternally inherited, permitting only maternal lineage identification and offering less discriminatory power than autosomal markers. Achieving forensic-grade profiles from compromised samples, therefore, requires the careful selection of target sequences that are short enough to permit amplification from degraded fragments while minimizing the risk of data misinterpretation.

In recent years, numerous iiSNP markers have been developed as a robust alternative for analyzing degraded DNA. Unlike STRs, which are defined by length variation in a repeated motif, a SNP represents a single base change at a specific nucleotide position within the genome [104]. This fundamental difference in structure is the source of their key advantages for the analysis of degraded forensic samples. A key advantage is that a relatively small panel of 50–60 SNP markers can achieve a discriminative power for differentiating unrelated individuals that is comparable to 12–15 STR markers [105,106]. SNP markers are short nuclear DNA segments located on autosomes, like STRs, but they are significantly shorter in length [107]. The shorter amplicon size of SNPs, typically averaging around 100 nucleotides, greatly facilitates PCR amplification from heavily fragmented DNA templates that would fail with longer STR assays [108,109]. Furthermore, forensic SNP panels are strategically designed to include markers with extremely short amplicons, often below 80 bp, specifically to target the smallest preserved fragments in a degraded sample [110]. Additionally, their biallelic nature (existence of only two possible alleles per locus) simplifies automation, data analysis, and interpretation compared to the multi-allelic and stutter-prone nature of STRs [20]. Multiple studies have consistently demonstrated that SNP markers provide superior and more reliable results than STRs when analyzing severely degraded DNA [108,111]. The analysis of SNPs in degraded DNA does not require prior knowledge of the individual’s genetic background. SNPs are abundant and widely distributed throughout the genome, providing numerous informative loci even in highly degraded samples, which makes them advantageous over STRs in forensic applications [112,113]. The presence of de novo SNPs in an individual does not significantly affect the reliability of SNP-based analysis, as the vast number of loci ensures sufficient markers are available for accurate identification [114,115]. Modern sequencing technologies and bioinformatics pipelines further allow the detection and interpretation of SNPs, mitigating potential issues from rare or novel variants [24]. Overall, SNP analysis provides a robust and reliable approach for forensic investigations, independent of prior genetic information.

Beyond iiSNPs, other specialized classes of SNPs provide investigative leads where STR typing is not possible. Phenotype-informative SNPs predict externally visible characteristics such as eye, hair, and skin color, providing intelligence in cases without a suspect [116]. Biogeographical ancestry SNPs offer information about the probable continental or regional ancestry of the sample donor, which can help focus investigations [117]. While these SNP types do not provide a unique identifier like iiSNPs, they are greatly valuable for generating leads and prioritizing suspects in otherwise cold cases. The ability to obtain both identity and phenotypic information from the same degraded sample using a single NGS platform represents a significant leap forward in forensic capabilities [118].

The advancement of NGS technologies has provided a monumental leap forward in the analysis of highly degraded DNA, offering significant advantages over conventional CE [18]. NGS platforms allow for the simultaneous examination of massive panels of SNP markers from autosomes, the X chromosome, and the Y chromosome, with dramatically higher sensitivity and throughput [21,22,62]. This rapid technological development has yielded numerous commercially available forensic SNP panels that enable the simultaneous analysis of hundreds of markers, generating an immense amount of genetic information from even minimal sample material [18,119]. This efficiency is paramount in forensic casework, as it conserves precious DNA extract for potential future reanalysis or confirmation testing. NGS also provides quantitative data (read counts) that can help detect mixtures and assess heterozygote balance more effectively than CE-based methods, adding a layer of analytical depth [120].

The utility of SNP analysis extends beyond identifying unknown individuals to significantly increasing the statistical likelihood in kinship testing when STR markers alone provide insufficient confidence to confirm an alleged biological relationship. A practical demonstration of this utility involved the analysis of two early medieval skeletons excavated from the Bled–Pristava archaeological site [95]. Prolonged postmortem intervals and environmental factors are known to severely impact DNA preservation in archaeological skeletons, leading to fragmentation and damage that often results in low STR typing success, as longer STR amplicons require sufficient DNA quality [121]. In case of non-adult skeletons, DNA is even less preserved than in adult skeletons, because bones are more porous and less dense [122]. Consequently, STR analysis could not confirm kinship between the two skeletons from the grave due to limited amplification success and low statistical support. The incorporation of the Precision ID Panel SNP markers successfully generated complete autosomal SNP profiles, yielding a sufficiently high statistical probability to confidently confirm a sister-sister relationship (Y-chromosomal SNP typing did not produce profiles). Independent anthropological and genetic analyses, including amelogenin testing, had confirmed that both skeletons were female. This case underscores the high number of analyzed SNP markers, ensuring a sufficient discriminative power to accurately establish kinship, even with highly fragmented DNA [108,111]. The Precision ID Identity Panel (Thermo Fisher Scientific), used in this research, allows for the simultaneous genotyping of 124 iiSNP markers—90 autosomal and 34 Y-chromosomal—producing unique genotypes highly suitable for identification. The extremely short length of the amplified fragments (averaging 132 bp for autosomal SNPs and 141 bp for Y-SNPs) enables successful typing of degraded DNA samples where standard STR typing has failed or yielded only partial results [123]. Critically, a complete analysis of all 124 SNP markers provides a higher discriminative power than the successful typing of a standard 15 STR marker panel. Furthermore, even partial SNP profiles obtained from low-quality or low-quantity DNA retain remarkably high discriminative power [124,125]. This allows for the reliable individualization of biological traces, and the identification of human remains in scenarios where STR typing fails or produces only poorly informative profiles, thereby solving cases that would otherwise remain unresolved [126]. The robustness of SNP analysis, combined with the power of NGS, is transforming the approach to the most challenging forensic samples.

## 6. Discussion

In life, DNA lesions are continuously corrected by repair mechanisms, but after death, these systems cease to function. Endogenous nucleases and autolytic enzymes released from lysed cells rapidly introduce strand breaks in the early post-mortem interval [36,37]. Hydrolytic processes, particularly depurination, cause basic sites and subsequent strand scission, resulting in progressive fragmentation [12]. Oxidative reactions continue to affect bases and sugar moieties even after death, either through residual reactive oxygen species or metal-catalyzed oxidants, leading to miscoding lesions and breaks [37,127]. Colonization of tissues and bone by microorganisms introduces exogenous nucleases and reactive metabolites that further damage DNA [127]. Environmental parameters such as temperature, humidity, pH, and exposure to UV or ionizing radiation strongly influence degradation rates, with warm, moist, acidic or UV-exposed conditions accelerating decay [38,47]. The microstructure of the substrate also plays an important role, as compact bone and teeth offer better protection than soft tissue [47]. Burial environment chemistry, including soil composition and salts, or immersion in seawater, additionally affects DNA stability [38]. Post-recovery treatments, such as chemical cleaning or harsh maceration procedures, can further reduce the quantity and quality of DNA [47]. Together, these processes shape the fragment length distribution and the characteristic miscoding patterns typical of degraded forensic and ancient samples [36].

Genetic markers used for analyses of forensic samples are STRs, iiSNPs, and mtDNA. STRs are the gold standard for forensic identification. They are highly polymorphic and discriminatory, supported by large databases and mature interpretation frameworks. Limitations include stutter artefacts and reduced success with degraded DNA [23]. Identity SNPs (iiSNPs) are biallelic markers with short amplicons, very suitable for degraded/low-template DNA and NGS workflows. They require large panels for discrimination comparable to STRs, and population allele frequency data and bioinformatics are critical [24,25,28]. MtDNA is a high copy number per cell, making it invaluable when nuclear DNA fails (e.g., hair shafts, ancient bones). It is useful for maternal lineage studies but offers limited individualizing power due to shared haplotypes and heteroplasmy [26].

The forensic genetic analysis of compromised biological samples is a multifaceted challenge that hinges on selecting the appropriate methodological approach based on the sample’s preservation state. The primary obstacle is the fragmented nature of degraded DNA, which severely impedes the amplification of longer STR amplicons, often resulting in partial or failed profiles from quantities below 100 pg [90,101]. While mtDNA has been a traditional recourse due to its high copy number [13,14,80], its limited discriminatory power has enabled the way for more robust solutions. The analysis of iiSNPs represents a critical advancement, as their shorter amplicon size and biallelic nature make them exceptionally suitable for profiling highly fragmented DNA while providing comparable discriminatory power to STRs from large panels [104,106,108,111].

The integration of NGS has been transformative, enabling the simultaneous, quantitative analysis of hundreds of SNP markers from minimal input DNA [18,21,22]. This capability allows for the conservation of precious extract, the generation of data from ultra-short fragments, and the concurrent analysis of iiSNPs with panels for biogeographical ancestry and phenotype prediction, thereby generating investigative leads from previously intractable samples [62,116,117,120].

However, these technological advancements are built upon the foundation of rigorous laboratory practice. The entire process—from evidence collection to analysis—relies on stringent anti-contamination protocols and optimized silica-based extraction methods designed to co-purify DNA while removing potent environmental inhibitors [32,60,61]. The role of accurate DNA quantification is paramount, as it provides the essential metrics—concentration, degradation index, and inhibitor presence—required to make an informed choice between STR and SNP workflows [81].

Despite significant advancements in NGS technologies, several challenges persist in forensic applications. Sequencing errors, including substitutions and indels, may compromise the accuracy of allele calls, particularly when analyzing degraded or low-template DNA [128]. Amplification bias remains a limitation, as PCR enrichment can lead to preferential amplification of certain alleles, resulting in allele drop-out or false homozygosity [129]. Furthermore, contamination is a critical issue: even trace levels of exogenous DNA can be amplified and sequenced, producing misleading or mixed profiles [130,131].

The interpretation of partial profiles is also challenging. Probabilistic genotyping frameworks and likelihood-based statistical models can assist in evaluating the probability of identity and assessing profiles affected by allelic drop-out, drop-in, or stutter artifacts [88,130]. Nevertheless, variation in library preparation, sequencing chemistries, and bioinformatic pipelines may affect reproducibility and cross-laboratory comparability [128,129].

From a practical perspective, the cost of analysis remains a significant factor in forensic casework. Traditional STR typing using CE is well-established, relatively inexpensive, and allows rapid generation of profiles from a moderate number of loci. In contrast, NGS analysis of iiSNP panels involves higher consumables and instrument costs, more complex library preparation, and extensive data processing, resulting in per-sample costs that are often 5–10 times higher than CE-based STR typing [129,131]. However, NGS offers substantially higher multiplexing capability, the ability to analyze degraded and low-template DNA, and the simultaneous recovery of biogeographical ancestry or phenotype-informative markers, potentially justifying the higher cost for complex or compromised samples [129].

Looking ahead, continued improvements in error-correction algorithms, molecular barcoding, and consensus sequence generation may reduce sequencing errors and amplification bias, while standardization of pipelines may improve reproducibility [129]. Nonetheless, even with technological advances, it is not always possible to recover a useful profile from highly degraded or limited DNA. Therefore, a case-by-case evaluation is essential, integrating biological context, sample quality metrics, cost considerations, and methodological limitations to ensure reliable and interpretable results [131].

The use of SNPs for predicting phenotype or biogeographical ancestry in forensic genetics raises important ethical and legal concerns. Predictive markers can reveal sensitive information about an individual, such as physical traits, disease susceptibility, or ancestral origins, which may not be relevant to the investigation [118] (Kayser & Schneider, 2009). Misuse or unauthorized access to such data could lead to discrimination, privacy violations, or stigmatization of individuals or populations. Legal frameworks governing forensic genetic analyses differ across jurisdictions, creating potential challenges in cross-border investigations and data sharing. Informed consent and clear policies on data retention, access, and reporting are essential to mitigate these risks. The interpretation of phenotype or ancestry predictions must also account for the probabilistic nature of these inferences to avoid overgeneralization or erroneous conclusions. Transparent communication of the limitations, potential biases, and societal implications of these methods is necessary for both forensic practitioners and the public. Ethical oversight committees and professional guidelines can provide critical guidance to ensure responsible application. Integrating these considerations into forensic workflows strengthens the legitimacy and societal acceptance of forensic DNA technologies. Ultimately, balancing investigative utility with respect for individual rights is crucial in the expanding use of SNP-based prediction practices [118].

## 7. Conclusions

In summary, the forensic genetic approach to the analysis of challenge samples has evolved to a sophisticated, sample-specific approach. The journey of analyzing degraded DNA is a story of moving from a reliance on a single technology to the intelligent and flexible application of a complementary genetic toolkit, where STR profiling, SNP genotyping, and NGS are viewed as synergistic rather than competing technologies [132].

This progression is fundamentally driven by a deep understanding of the complex, dynamic process of DNA degradation itself. The irreversible fragmentation and loss of genetic information caused by environmental factors necessitate this adaptable strategy. The future of the field will be shaped by the ongoing development of even more sensitive assays, robust bioinformatic tools for interpreting complex NGS data, and critical international standardization efforts [133].

This continuous synergy between biochemistry, technology, and informed interpretation ensures that the evidentiary value of biological material will continue to be reliably unlocked from the most challenging samples encountered in both crime scene trace samples and samples collected from unknown human remains, long after the process of degradation has begun.

The forensic DNA analysis field is increasingly recognizing the necessity for global standardization to ensure consistency and comparability across laboratories worldwide. Such standardization efforts are crucial for harmonizing protocols, quality control measures, and data interpretation criteria, thereby enhancing the reliability of forensic findings. Additionally, the integration of interdisciplinary approaches is becoming essential, with collaborations between forensic scientists, biochemists, and bioinformaticians facilitating the development of more robust analytical techniques. Incorporating complementary biomolecular methods, such as proteomics and metabolomics, can provide a more comprehensive understanding of degraded samples, potentially revealing information that DNA analysis alone cannot. Furthermore, advancements in artificial intelligence and machine learning are poised to revolutionize data analysis in forensic genomics, enabling more accurate and efficient interpretation of complex datasets. The adoption of these technologies requires careful consideration of ethical implications, particularly concerning data privacy and the potential for misuse. International collaborations and data-sharing initiatives are vital to overcome the challenges posed by resource disparities among forensic laboratories. Moreover, continuous education and training programs are necessary to equip forensic professionals with the skills needed to implement and adapt to these evolving methodologies. The establishment of global databases and repositories can further support standardization efforts and facilitate the exchange of knowledge and resources. Ultimately, these combined efforts will enhance the efficacy and equity of forensic DNA analysis worldwide, ensuring justice is served through scientifically sound and universally accepted practices [21].

## Figures and Tables

**Table 1:** Comparison of forensic genetic markers: STRs, iiSNPs, and mtDNA [23,24,25,26,27,28].

Parameter	STRs	iiSNPs	mtDNA
Copy number per cell	Single-copy nuclear loci (2 copies per diploid cell per autosomal locus) [23]	Single-copy nuclear loci (2 copies per diploid cell for autosomal SNPs) [24,25]	High copy number (hundreds–thousands per cell) [26,27]
Amplicon size	Typically, 100–500 bp; longer amplicons limit degraded DNA use [23,27]	Very short (<150 bp); suitable for degraded DNA, NGS-optimized [24,25]	Small overlapping amplicons (<200 bp) or whole mitogenome panels [26]
Discriminative power	Very high (RMP ~10^−15^ to 10^−20^) [23]	Moderate per locus; needs large panels (90–120 SNPs, RMP ~10^−34^) [25,28]	Lower individualization, shared haplotypes [26]
Mode of inheritance	Autosomal, biparental, codominant [23]	Autosomal, biparental; Y-SNPs paternal [24]	Maternal inheritance, haploid, non-recombining [26]
Features	Multi-allelic, stutter artefacts, well-established CE workflows [23]	Biallelic, low mutation rate, minimal stutter, scalable in NGS [24,25]	Circular 16.6 kb genome, higher mutation rate, heteroplasmy [26]
Advantages	Extremely high discrimination; mature interpretation framework [23]	Short amplicons, good for degraded/low-template DNA [24,28]	Useful when nuclear DNA fails (hair shafts, bones); lineage tracing, recovery from degraded/low quantity samples [26]
Limitations	Poor performance with degraded DNA; stutter complicates mixtures [23]	Less informative per locus; requires large panels, bioinformatics, and higher costs [24,28]	Lower discrimination; heteroplasmy and NUMTs complicate analysis [26]
Typical forensic use	Routine human ID, databases, kinship [23]	Degraded/low-quantity samples; supplementary to STRs [25,28]	Maternal lineage, degraded remains, ancient samples [26]

**Table 2:** Summary of main degradation factors influencing DNA preservation [8,40,41,42,43,47,48,49,50,51,52,53,54,55,56,57,58,59].

Degradation Factor	Effect on DNA	Practical Implications
Temperature	Primary regulator of chemical and microbial activity; higher temperatures accelerate hydrolysis and oxidation; 2 °C rise can measurably destabilize DNA, 10 °C rise can double or triple degradation rates [47,48].	Cold and constant environments (e.g., permafrost) preserve DNA exceptionally well, while hot and humid climates cause near-total degradation [49,50].
Moisture	Essential for hydrolysis and microbial growth; high water activity is extremely detrimental to DNA [8,51].	Dry depositional contexts favor preservation; waterlogged or humid conditions accelerate decay.
pH	Strongly acidic or alkaline soils catalyze degradation; neutral to slightly alkaline conditions suppress nuclease activity [8,51].	Acidic soils accelerate soft tissue decomposition up to three times faster than alkaline soils [52].
Soil/sediment geochemistry	DNA can adsorb to clays and hydroxyapatite, providing protection [53]; organic-rich soils enhance microbial activity and release PCR inhibitors such as humic acids [54,55].	Mineral-rich soils promote preservation; organic soils hinder both preservation and downstream DNA analysis.
Microbial activity	Direct enzymatic cleavage of DNA; indirect damage via acidification and alteration of microenvironment [56].	Microbial abundance must be considered; high activity drastically reduces DNA recovery.
Oxygen availability	Promotes oxidative damage via free radical formation [57].	Anaerobic conditions are more favorable for long-term preservation.
Physical forces	Freeze–thaw cycles, sediment pressure, and plant root growth shear DNA into shorter fragments [57,58].	Deeper burial reduces such forces and provides environmental buffering.
Burial depth	Deeper layers buffer temperature, reduce oxygen exposure, and eliminate UV radiation [8,59].	Deep burial contexts are generally more favorable for long-term DNA survival.
Duration of exposure	Longer exposure time generally accelerates and intensifies DNA degradation [40,41,42].	Older remains typically yield lower DNA quality and quantity; rapid recovery and analysis are advantageous.
Post-recovery handling	Improper collection or storage introduces modern contamination or further damages DNA [43].	Strict contamination control and standardized protocols are required during sampling, storage, and analysis.

**Table 3:** Summary of calculation methods for STR markers [4,84,85,87,88,89,90].

Aspect	Description	References
Discriminatory power	The probability of two unrelated individuals sharing a full STR profile can be <1 in 1 billion.	[4,84,85]
Probability of Identity (PID)	Estimates the chance that two unrelated individuals will share an identical multilocus genotype. Typical values: <10^−15^ when 15–24 loci are analyzed.	[88]
Probability of Identity among siblings (PIDsib)	More conservative measures account for increased allele sharing among siblings. Typical values: 10^−4^–10^−6^.	[87,88]
Likelihood Ratio (LR)	It indicates how much more likely the sample belongs to the target individual than to a randomly selected person.	[89,90]

**Table 4:** Approximate Likelihood Ratio (LR) values for 16–24 STR loci [89].

Number of STR loci	LR for Unrelated Individuals	LR for Siblings (Close Relatives)
16	≈10^12^–10^13^	≈10^2^–10^3^
18	≈10^13^–10^14^	≈10^3^–10^4^
20	≈10^14^–10^15^	≈10^3^–10^5^
24	≈10^15^–10^16^	≈10^4^–10^6^

## Data Availability

No new data were created or analyzed in this study. Data sharing is not applicable to this article.
